# Tidal Volume in Idiopathic Pulmonary Fibrosis: Restriction Severity and Prognostic Information beyond Forced Vital Capacity

**DOI:** 10.1159/000553631

**Published:** 2026-09-04

**Authors:** Ekaterina Krauss, Silke Tello, Til K. Spreuer, Katharina Wendlandt, Ayca Tunay, Poornima Mahavadi, Carlo Vancheri, Maria Molina Molina, Bruno Crestani, Andreas Guenther

**Affiliations:** a European IPF/ILD Registry and Biobank (eurIPFreg/bank, eurILDreg/bank), Giessen, Germany; b Center for Interstitial and Rare Lung Diseases, Universities of Giessen and Marburg Lung Center (UGMLC), Justus-Liebig-University Giessen, Member of the German Center for Lung Research (DZL), Giessen, Germany; c Cardio-Pulmonary Institute (CPI), Giessen, Germany; d Institute for Lung Health (ILH), Giessen, Germany; e Agaplesion Lung Clinic, Evangelisches Krankenhaus Mittelhessen, Giessen, Germany; f Department of Clinical and Experimental Medicine, Regional Referral Center for Rare Lung Diseases, University Hospital Policlinico, University of Catania, Catania, Italy; g ILD Unit, Respiratory Department, University Hospital of Bellvitge, IDIBELL, University of Barcelona, Barcelona, Spain; h Service de Pneumologie, Allergologie et Transplantation, AP-HP, Hôpital Bichat, Paris, France

**Keywords:** Idiopathic pulmonary fibrosis, Tidal volume, Tidal breathing, European registry for idiopathic pulmonary fibrosis, European registry for interstitial lung diseases

## Abstract

**Introduction:**

Idiopathic pulmonary fibrosis (IPF) is characterised by progressive loss of lung compliance, which raises the elastic work of breathing and alters resting ventilatory strategy. Clinical assessment of IPF still relies on forced manoeuvres, particularly forced vital capacity (FVC), which capture maximal performance rather than this resting adaptation. How tidal volume (VT) evolves over the IPF course is not well characterised. VT and the VT/FVC ratio offer an effort-independent window onto the same physiology, characterised here longitudinally.

**Methods:**

We analysed 613 IPF patients enrolled at 3 European eurIPFreg centres (Giessen, Barcelona, Paris) between 2009 and 2025. Baseline VT, FVC, and VT/FVC were compared between survivors and non-survivors. Transplant-free survival was analysed by Cox regression, including age, sex, antifibrotic treatment, log-transformed VT and FVC, and their interaction. Discrimination was assessed by Harrell’s C index with 20-fold cross-validation and time-dependent AUCs. Longitudinal trajectories were modeled over 60 months.

**Results:**

VT followed a dynamic, non-monotonic trajectory consistent with progressive ventilatory adaptation, rising transiently to ∼5% above baseline at months 18–21, coincident with the steepest segment of FVC decline (months: 6–18), and returning slowly toward baseline thereafter. The VT/FVC ratio rose continuously (+17% at 60 months), the only marker to evolve monotonically, while FVC declined progressively (−8.8%). Higher baseline VT showed a trend toward improved survival (log-rank *p* = 0.092; hazard ratio [HR]: 0.88, 95% CI: 0.75–1.04), and the VT/FVC ratio stratified survival significantly (log-rank *p* < 0.001), as did baseline FVC (HR: 0.59 per unit log-FVC, 95% CI: 0.49–0.69; *p* < 0.001). The VT × FVC interaction approached significance (HR: 1.12, 95% CI: 0.98–1.30; *p* = 0.08), suggesting a joint physiological signal. Antifibrotic therapy reduced mortality (HR: 0.63, 95% CI: 0.50–0.81; *p* < 0.001).

**Conclusion:**

VT in IPF follows a dynamic, non-monotonic course, rising during the steepest phase of FVC decline and returning toward baseline thereafter, consistent with a ventilatory adaptation in which increased VT serves as a transient lower cost strategy for maintaining minute ventilation before the elastic load makes it unsustainable. The VT/FVC ratio integrates this rising drive with falling vital capacity, evolves monotonically, and stratifies survival, identifying it as an effort-independent physiological descriptor of restrictive remodelling. Together, VT and VT/FVC enrich the standard FVC-based assessment of IPF by capturing the resting ventilatory dimension of disease progression.

## Introduction

Idiopathic pulmonary fibrosis (IPF) is characterised by progressive stiffening of the lung parenchyma, which increases the elastic load and gradually diminishes ventilatory reserve [1, 2]. These structural changes reduce lung compliance, leading to a higher transpulmonary pressure gradient and an increased effort required to achieve a tidal volume [3]. Consequently, patients adjust their breathing patterns well before overt declines in conventional forced expiratory measures are detectable, underscoring a disconnect between early mechanical impairment and standard physiological markers [4].

Despite these early mechanical alterations, clinical monitoring relies largely on forced manoeuvres such as forced vital capacity (FVC), which reflects maximal performance but provides limited insight into resting ventilation [5]. Patients may adopt altered breathing patterns, reduce tidal excursion, or shift their operating volume range long before measurable declines appear in spirometric indices, signalling an unmet need for markers that describe everyday breathing behaviour rather than maximal forced effort [6].

Tidal volume (VT) represents the fundamental operating volume of the respiratory system during spontaneous breathing [7]. It integrates multiple determinants, including lung compliance, inspiratory muscle function, neuromechanical drive, and behavioural adaptation, but has received comparatively little attention in the clinical assessment of IPF [8]. There is therefore a gap in understanding how restrictive mechanics influence the volumes patients actually use during everyday breathing [9].

The ratio of VT to FVC offers an intuitive, effort-independent descriptor of individual breathing behaviour by relating operating volume to remaining structural capacity. A higher VT/FVC ratio indicates that a larger fraction of the available lung volume is recruited with each tidal breath, suggesting reduced ventilatory reserve and greater mechanical demand to sustain adequate ventilation. Conversely, lower VT/FVC ratios reflect breathing well below structural limits, consistent with more conservative ventilatory strategies.

Physiologically, these patterns may represent alternative adaptations to progressive restriction. In the setting of markedly reduced FVC, patients face a fundamental trade-off: maintaining VT at the cost of increased work of breathing, respiratory muscle load, and mechanical stress, or reducing VT to limit effort and stress at the expense of less efficient alveolar ventilation and permissive hypercapnia. A higher VT/FVC ratio may therefore reflect an active strategy to preserve alveolar ventilation despite rising elastic load, whereas a lower ratio may indicate acceptance of reduced ventilation to minimise mechanical and muscular strain.

Importantly, these adaptive responses are not inherently maladaptive. Breathing at higher VT/FVC ratios may increase respiratory effort, dyspnoea, and vulnerability to mechanical injury, while persistently low ratios may compromise gas exchange when ventilatory reserve becomes critically limited. Thus, VT/FVC does not represent a simple linear marker of disease severity but rather captures how patients operate within a shrinking ventilatory space.

Although preliminary observations suggest that VT/FVC varies across the spectrum of physiological impairment, it has remained unclear whether this variability reflects a clinically meaningful dimension of disease behaviour or merely parallels established markers of restriction such as FVC. Until now, the physiological and prognostic relevance of VT/FVC has not been systematically evaluated in a large, real-world IPF cohort [6, 10].

We sought to evaluate whether tidal-breathing parameters, specifically VT and VT/FVC, correlate with established severity indices, track meaningful changes over time, and provide prognostic information beyond FVC and gas-exchange markers. Using data from the multicentre European IPF registry, we examined the cross-sectional associations, longitudinal dynamics, and survival relationships of these measures, directly comparing them with established predictors to assess their independent clinical value in IPF.

## Study Objectives

We examined the cross-sectional associations of VT and VT/FVC with established functional measures, evaluated their longitudinal trajectories, and assessed their relationship with survival using multivariable Cox regression, model discrimination, and comparative performance metrics to quantify their added clinical value.

## Materials and Methods

### Study Design and Patient Cohort

This study included 613 patients with IPF from 3 centres of the eurIPFreg: Giessen (*n* = 192), Barcelona (*n* = 154), and Paris (*n* = 267). All patients were enrolled in the European IPF Registry (eurIPFreg) or its continuation platform, the European ILD Registry (eurILDreg) between November 2009 and October 2025 [11]. Both registries operate under harmonised protocols, are ethically approved (Justus-Liebig-University Giessen, AZ 111/08), and are registered in international clinical trial databases (ClinicalTrials.gov NCT02951416; DRKS00028968). Written informed consent was obtained from all participants [11, 12]. IPF diagnoses were established through multidisciplinary discussion according to ATS/ERS/JRS/ALAT guidelines [2]. For this analysis, baseline was defined as the first spirometry within the registry at which simultaneous VT and FVC measurements were available, which were required to calculate the VT/FVC ratio.

### Measurements and Outcomes

To comprehensively evaluate patient status, multiple standardised assessments were performed as part of routine clinical care. Demographic and clinical data included age, sex, body mass index (BMI), smoking history (status and pack-years), and antifibrotic therapy (pirfenidone or nintedanib), with treatment duration extracted from structured registry entries. Patients were considered treated if they had received the same antifibrotic for at least 6 months. Clinical information was obtained from medical records and standardised registry questionnaires, with any uncertainties clarified through review of physician documentation and medication records. Pulmonary function tests (PFTs) were conducted in accordance with internationally standardised protocols [13–15]. Parameters assessed during PFTs included FVC, VT, and the derived ratio (VT/FVC).

VT was recorded during quiet, resting tidal breathing in the seated position, after a stabilisation period of at least 1 min, and was reported as the mean of the last 3 regular tidal breaths obtained during body plethysmography. The VT/FVC ratio was calculated from these paired values. The primary endpoint was a composite of all-cause death or lung transplantation.

### Statistical Analysis

All analyses were performed in R. Continuous variables are reported as mean ± SD or median (IQR), as appropriate, and categorical variables as *n* (%). Baseline characteristics were compared between survivors and non-survivors using Student’s *t* tests, Wilcoxon rank-sum tests, or chi-square tests, as appropriate. Associations between VT/FVC and other continuous variables were assessed using Pearson correlation.

The primary outcome was a composite of death or lung transplantation. Survival time was measured from the baseline assessment to the event of interest or to last follow-up; patients alive without transplantation at last contact were right-censored. Survival was analysed by Cox proportional hazards regression, with hazard ratios (HRs) reported alongside 95% confidence intervals. Pre-specified models comprised a base model adjusted for age, sex, and antifibrotic treatment, separate models adding FVC or VT, a combined model including both FVC and VT, and an extended model including the VT × FVC interaction. VT and FVC were log-transformed and standardised due to their skewed distributions; age and the categorical variables were entered without transformation. Continuous values of VT and FVC were retained in all Cox models to preserve statistical power and avoid arbitrary cutoffs. Model discrimination was quantified using Harrell’s C-index, with 20-fold cross-validation applied to all Cox models to limit overfitting; cross-validated and apparent concordance were compared. Time-dependent discrimination was further assessed by calculating AUCs at 1, 3, 6, 12, 24, 36, 48, and 60 months from Cox-derived risk estimates. A randomly generated variable served as a negative control.

For visualisation only, patients were stratified into quantiles of baseline VT, FVC, and VT/FVC, and survival differences across strata were displayed by Kaplan-Meier curves and compared by log-rank test. Quantile stratification was used to ensure balanced group sizes and to facilitate visual assessment of graded survival differences.

Longitudinal analyses examined repeated measures of VT, FVC, and VT/FVC, with each measurement normalised to the individual patient’s baseline value. To avoid disproportionate influence of densely sampled individuals, repeated measurements were first averaged within monthly time points at the patient level, and population-level trends were then summarised using locally estimated scatterplot smoothing applied to the aggregated monthly values. All trajectories were anchored at unity at baseline by construction; consequently, the *t*_0_ point is the only time point without an associated 95% confidence interval. Confidence intervals at subsequent time points reflect between-patient variability within each monthly bin. All tests were two-sided, with *p* < 0.05 considered statistically significant.

## Results

### Baseline Characteristics

A total of 613 patients with IPF were included; 272 (44%) died or underwent lung transplantation during follow-up. Baseline characteristics are shown in Table 1. Survivors and non-survivors did not differ in age (median: 70 vs. 71 years; *p* = 0.13), BMI (28 vs. 27 kg/m^2^; *p* = 0.18), or baseline VT (0.85 vs. 0.84 L; *p* = 0.5). The proportion of male patients was numerically higher among non-survivors (81% vs. 74%; *p* = 0.067). Non-survivors had a lower FVC (2.3 vs. 2.7 L; *p* < 0.001), a higher VT/FVC ratio (0.37 vs. 0.34; *p* = 0.002), and a higher GAP index (5.0 vs. 3.9; *p* < 0.001), consistent with more advanced disease at baseline. Antifibrotic therapy for at least 6 months was less frequent among non-survivors (57% vs. 67%; *p* = 0.012), and median exposure to antifibrotic therapy was shorter (24 vs. 36 months; *p* < 0.001).

**Table 1.** Demographics, physiology, lung function, and treatment at baseline in the study cohort

Variable	Alive (*n* = 341)	Deceased (*n* = 272)	*p* value
Age, years	70 (63–76)	71 (62–77)	0.13
Male sex	252 (74%)	219 (81%)	0.067
BMI, kg/m^2^	28 (25–30)	27 (25–30)	0.18
Smoker (current/former)	121 (35%)	116 (43%)	0.084
Pack-years	22 (13–38) (*n* = 63)	29 (10–40) (*n* = 47)	0.30
GAP index	3.9±1.5 (*n* = 94)	5.0±1.5 (*n* = 66)	<0.001
Follow-up time, months	23 (6.2–48)	18 (9–33)	0.095
pO_2_, mm Hg	72 (65–77) (*n* = 83)	68 (59–74) (*n* = 69)	0.021
pCO_2_, mm Hg	38 (36–40) (*n* = 82)	38 (35–40) (*n* = 69)	0.40
pH	7.40 (7.40–7.42) (*n* = 77)	7.42 (7.40–7.45) (*n* = 69)	0.005
DLco (% predicted)	63 (50–74) (*n* = 103)	46 (36–59) (*n* = 75)	<0.001
FVC, L	2.7 (2.0–3.3)	2.3 (1.8–2.9)	<0.001
VT, L	0.85 (0.65–1.10)	0.84 (0.67–1.00)	0.50
VT/FVC	0.34 (0.26–0.43)	0.37 (0.30–0.46)	0.002
Antifibrotic therapy ≥6 months, *n* (%)	229 (67%)	155 (57%)	0.012
Duration of antifibrotic therapy, months	36 (21–54) (*n* = 229)	24 (15–43) (*n* = 155)	<0.001

Unless otherwise specified, parameters have no missing values; the number of patients with available data is given in parentheses. Values are reported as *n* (%) for categorical variables and as median (25th–75th percentile) or mean ± SD for continuous variables, as appropriate. *p* values were derived from the chi-square test for categorical variables, the Wilcoxon rank-sum test for non-normally distributed continuous variables, and Student’s *t* test for normally distributed continuous variables.

BMI, body mass index; DLco, diffusing capacity of the lung for carbon monoxide; FVC, forced vital capacity; GAP, Gender-Age-Physiology index; pCO_2_, arterial partial pressure of carbon dioxide; pO_2_, arterial partial pressure of oxygen; SD, standard deviation; VT, tidal volume; VT/FVC, ratio of tidal volume to forced vital capacity.

### Longitudinal Trajectories of Ventilatory Parameters

**Fig. 1.**
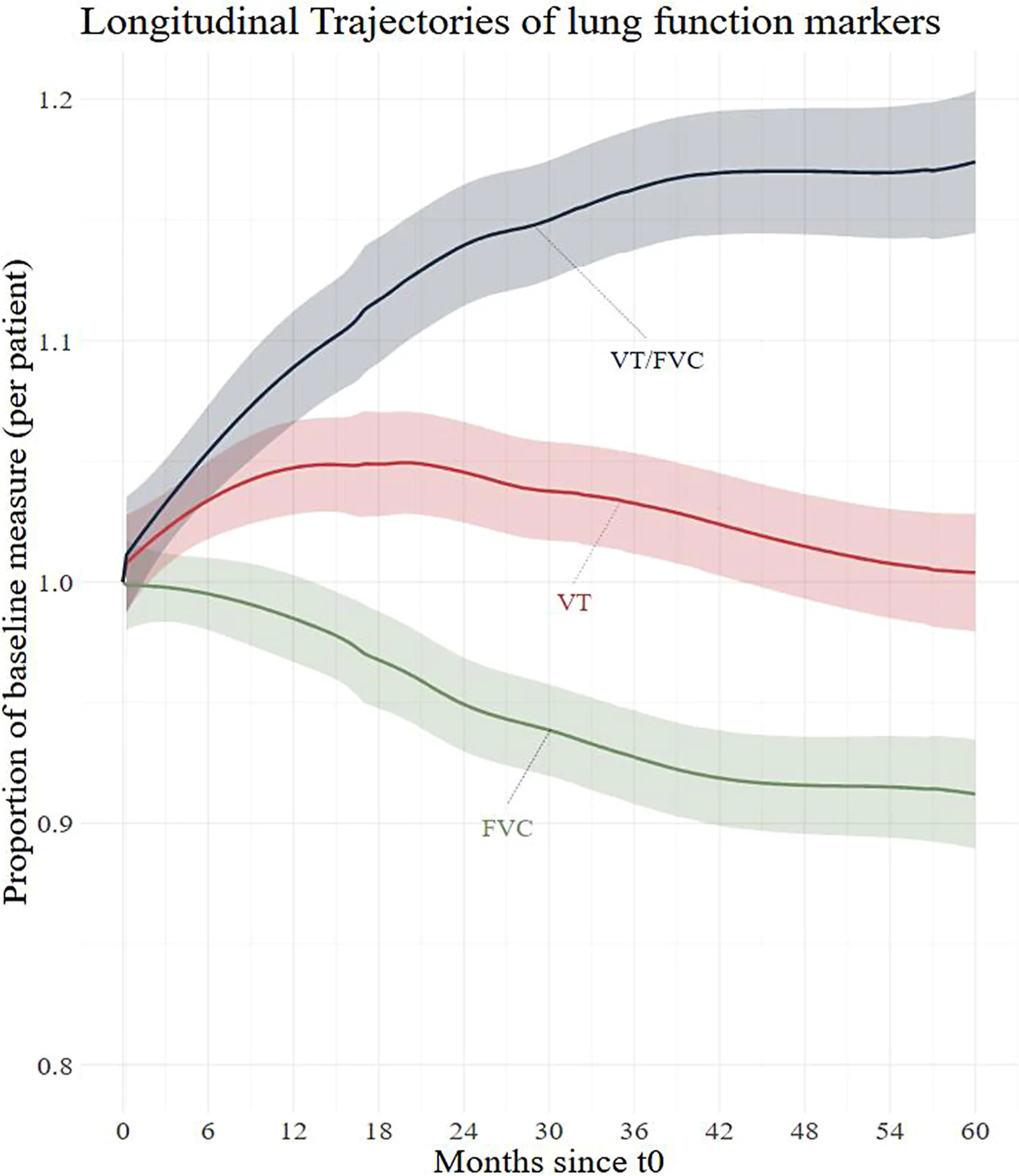
Longitudinal trajectories of tidal volume, forced vital capacity, and the VT/FVC ratio over 60 months. All values are expressed as a proportion of each patient’s baseline measurement (*t*_0_ = 1.0, anchored by definition). Repeated measurements were averaged within monthly bins at the patient level; population-level trajectories were estimated by locally estimated scatterplot smoothing (LOESS) applied to the binned per-patient means. Shaded bands represent 95% confidence intervals at each post-baseline time point. FVC declined progressively from the earliest follow-up, VT increased transiently during the first year before returning toward baseline, and VT/FVC rose continuously throughout follow-up. FVC, forced vital capacity; VT, tidal volume; VT/FVC, ratio of tidal volume to forced vital capacity.

Longitudinal trajectories of VT, FVC, and the VT/FVC ratio were analysed over 60 months of follow-up, with all values normalised to each patient’s baseline measurement (*t*_0_ = 1.0), as shown in Table 2 and Figure 1. FVC declined progressively from baseline, reaching −8.8% at 60 months. VT showed a different pattern: it increased modestly during the first 12 months (+4.7% at month 12) and then attenuated, returning toward baseline by month 60. The VT/FVC ratio rose continuously throughout follow-up, reaching +17% at 60 months. The number of patients contributing measurements declined from 183 at month 12 to 33 at month 60, and trajectories at later time points should therefore be interpreted with caution.

**Table 2.** Longitudinal change in forced vital capacity, tidal volume, and the VT/FVC ratio over 60 months of follow-up

Measure	Month 1	Month 3	Month 6	Month 12	Month 24	Month 36	Month 48	Month 60
FVC, Δ% from baseline	−0.1	−0.2	−0.5	−1.5	−5.1	−7.3	−8.4	−8.8
VT, Δ% from baseline	+1.2	+2.2	+3.4	+4.7	+4.5	+3.2	+1.5	+0.4
VT/FVC, Δ% from baseline	+1.7	+3.3	+5.4	+8.9	+14.0	+16.0	+17.0	+17.4
Patients, *n*	87	147	132	183	108	75	42	33

Values represent the percent deviation from each patient’s individual baseline measurement (*t*_0_ = 0%), aggregated within monthly time-point bins after per-patient averaging. Values are descriptive. Note the substantial decline in the number of contributing patients at later time points; trajectories beyond 36 months should be interpreted accordingly.

FVC, forced vital capacity; VT, tidal volume; VT/FVC, ratio of tidal volume to forced vital capacity; Δ%, percent change from each patient’s baseline value.

### Survival Modelling

Given the observed distribution of baseline respiratory parameters and their associations with survival, we next evaluated the prognostic significance of VT, FVC, and the VT/FVC ratio within a multivariable time-to-event framework. Survival modelling was undertaken to quantify these relationships while accounting for relevant clinical covariates and to assess whether baseline respiratory function independently predicted mortality risk.

**Fig. 2.**
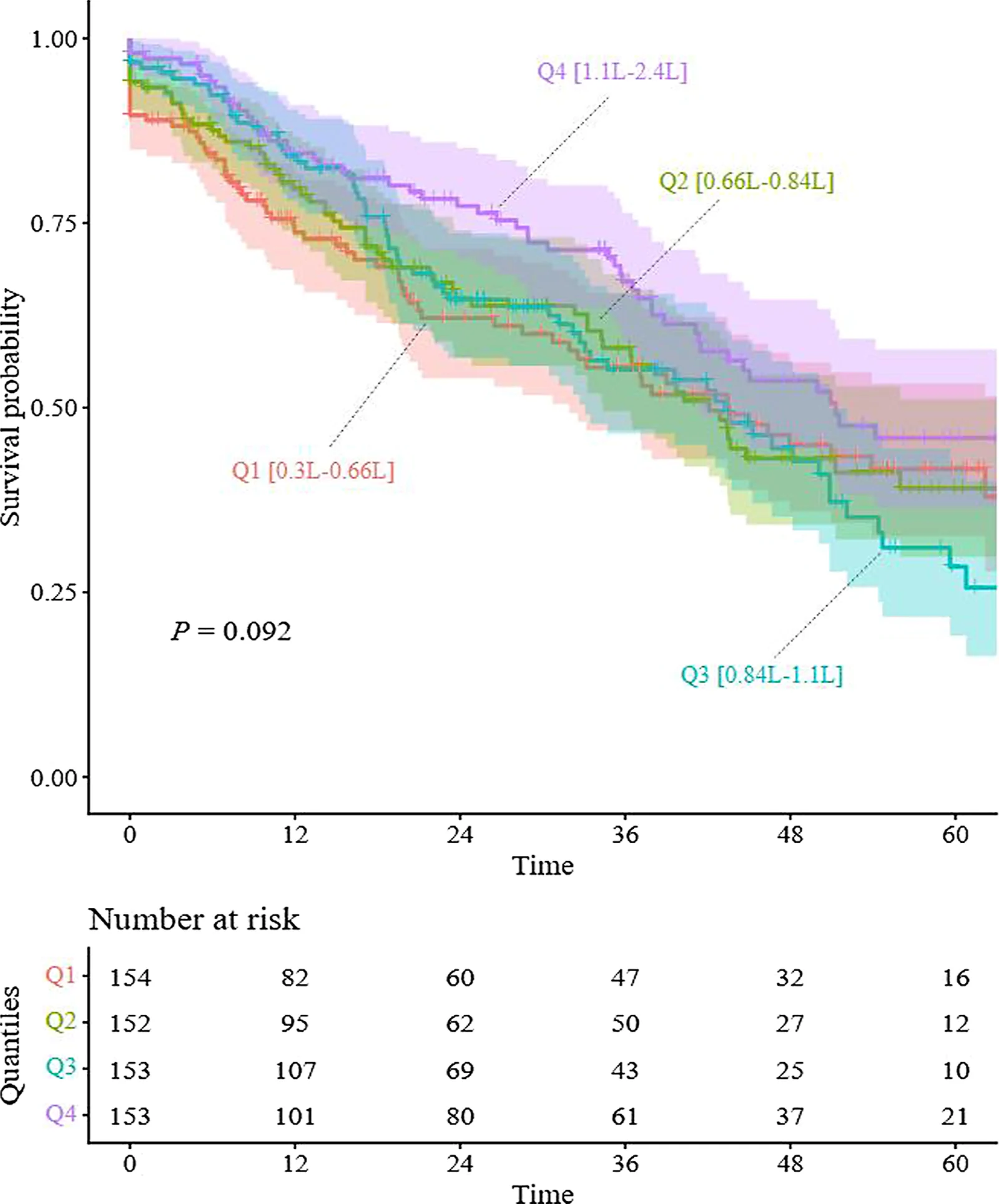
Association of baseline tidal volume (in litres) with all-cause mortality in idiopathic pulmonary fibrosis (*p* = 0.092). Patients were stratified into quantiles based on baseline VT values. Kaplan-Meier survival curves were compared using the log-rank test; survival differences across VT quantiles did not reach statistical significance. Q1–Q4, quantiles 1 through 4; VT, tidal volume.

**Fig. 3.**
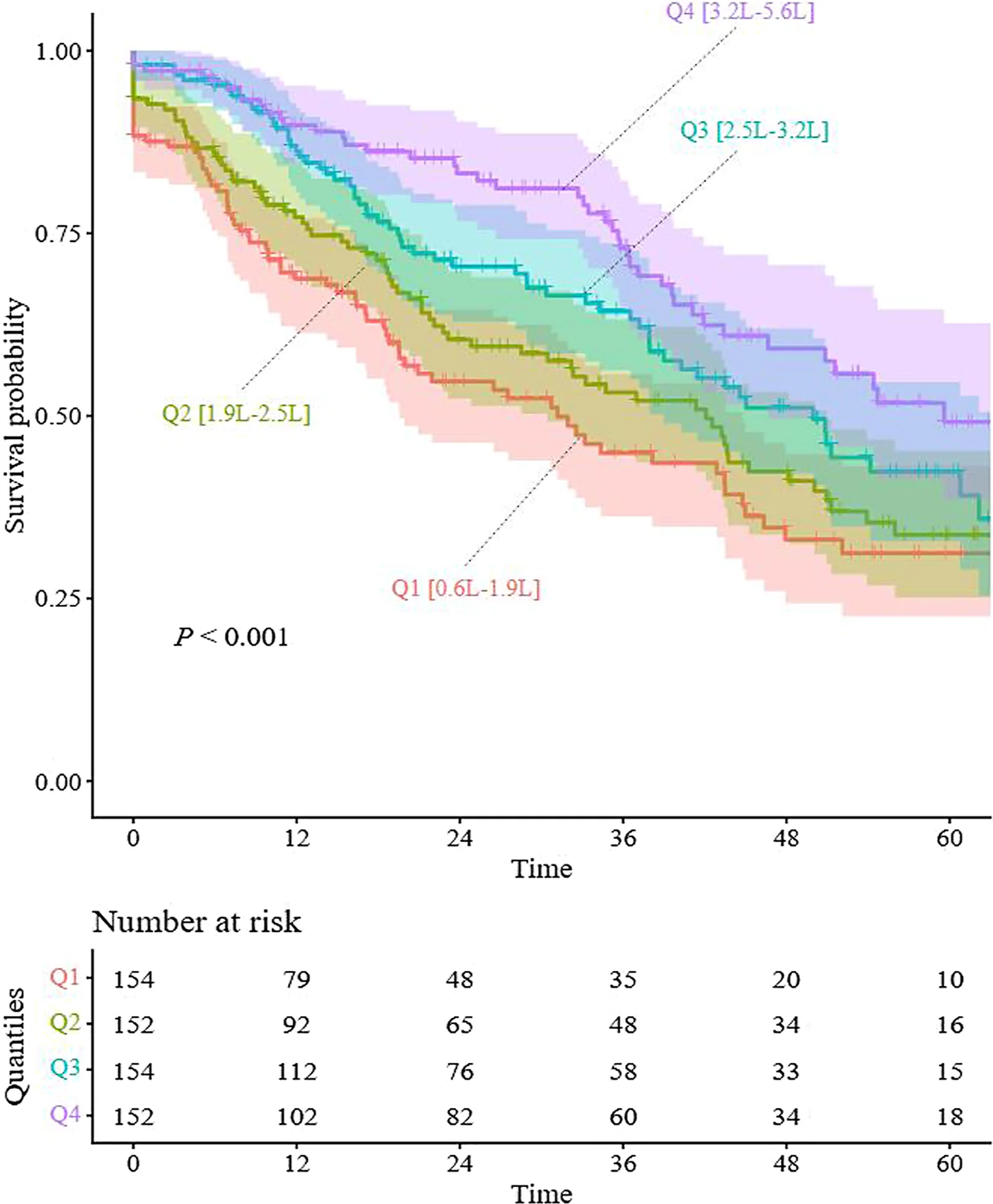
Association of baseline forced vital capacity (in litres) with all-cause mortality in idiopathic pulmonary fibrosis (*p* < 0.001). Patients were stratified into quantiles based on baseline FVC. Kaplan-Meier survival curves illustrate graded differences in mortality across groups, assessed using the log-rank test. FVC, forced vital capacity; Q1–Q4, quantiles 1 through 4.

**Fig. 4.**
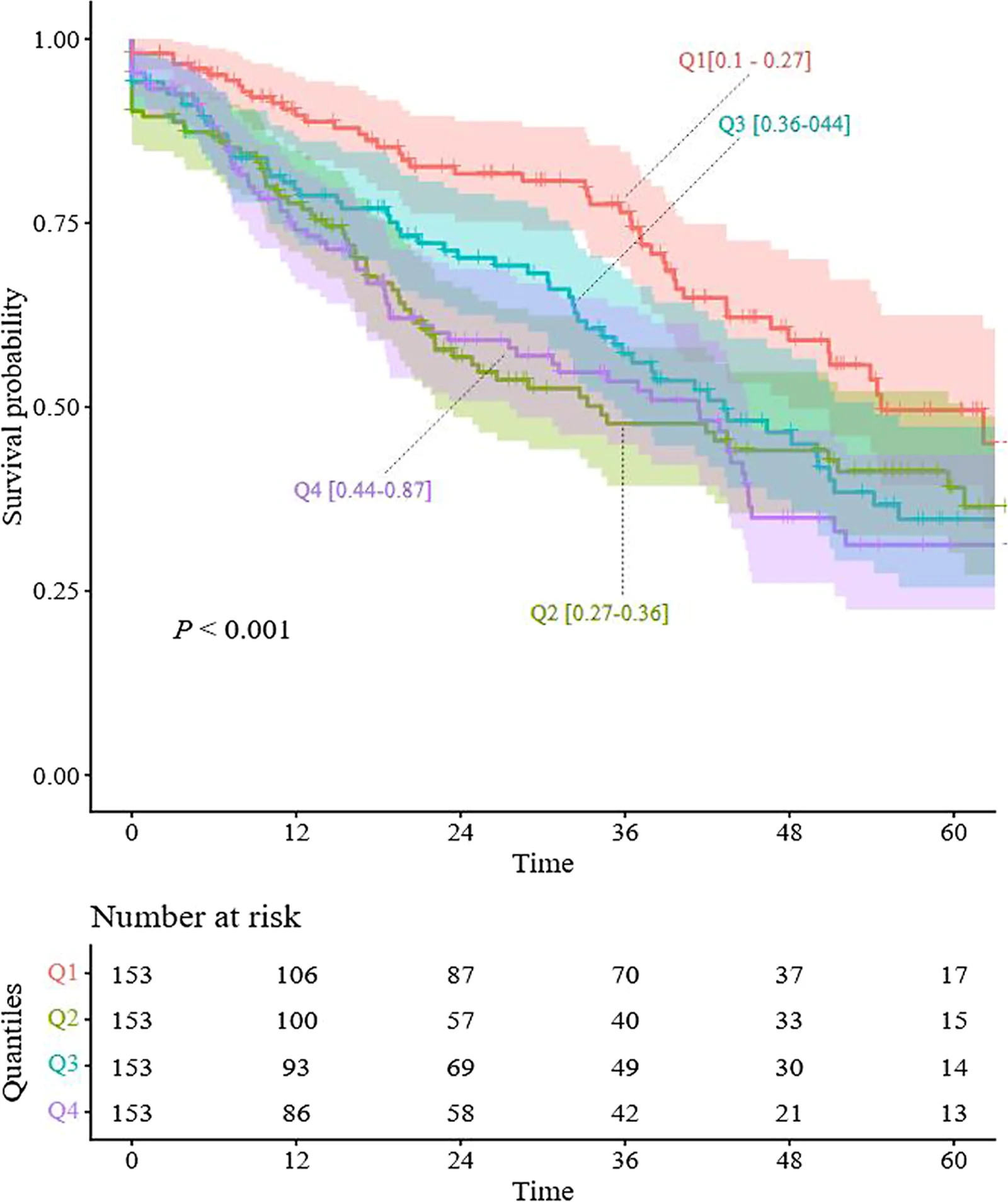
Association of the baseline VT/FVC ratio with all-cause mortality in idiopathic pulmonary fibrosis (*p* < 0.001). Patients were stratified into quantiles based on the baseline tidal volume-to-forced vital capacity ratio (VT/FVC). Kaplan-Meier survival curves show trends across quantiles, with higher VT/FVC ratios tending toward reduced survival. Differences between quantiles were assessed using the log-rank test. FVC, forced vital capacity; Q1–Q4, quantiles 1 through 4; VT, tidal volume; VT/FVC, ratio of tidal volume to forced vital capacity.

Figures 2–4 illustrate the association between baseline respiratory parameters and survival, visualised using Kaplan-Meier curves stratified by quantiles of VT, FVC, and VT/FVC, with log-rank tests for between-group comparison. For the cohort overall, median transplant-free survival was approximately 18 months (95% CI: 15–20). For VT, higher VT strata trended toward more favourable survival, although the difference across quantiles did not reach statistical significance (log-rank *p* = 0.092). FVC stratification revealed a clear separation of survival curves (log-rank *p* < 0.001), with lower baseline FVC linked to markedly worse survival and higher FVC strata associated with better outcomes. Survival patterns by VT/FVC ratio reflected the combined influence of VT and lung capacity, with graded differences across quantiles (log-rank *p* < 0.001).

### The Multivariable Cox Proportional Hazards Model

**Fig. 5.**
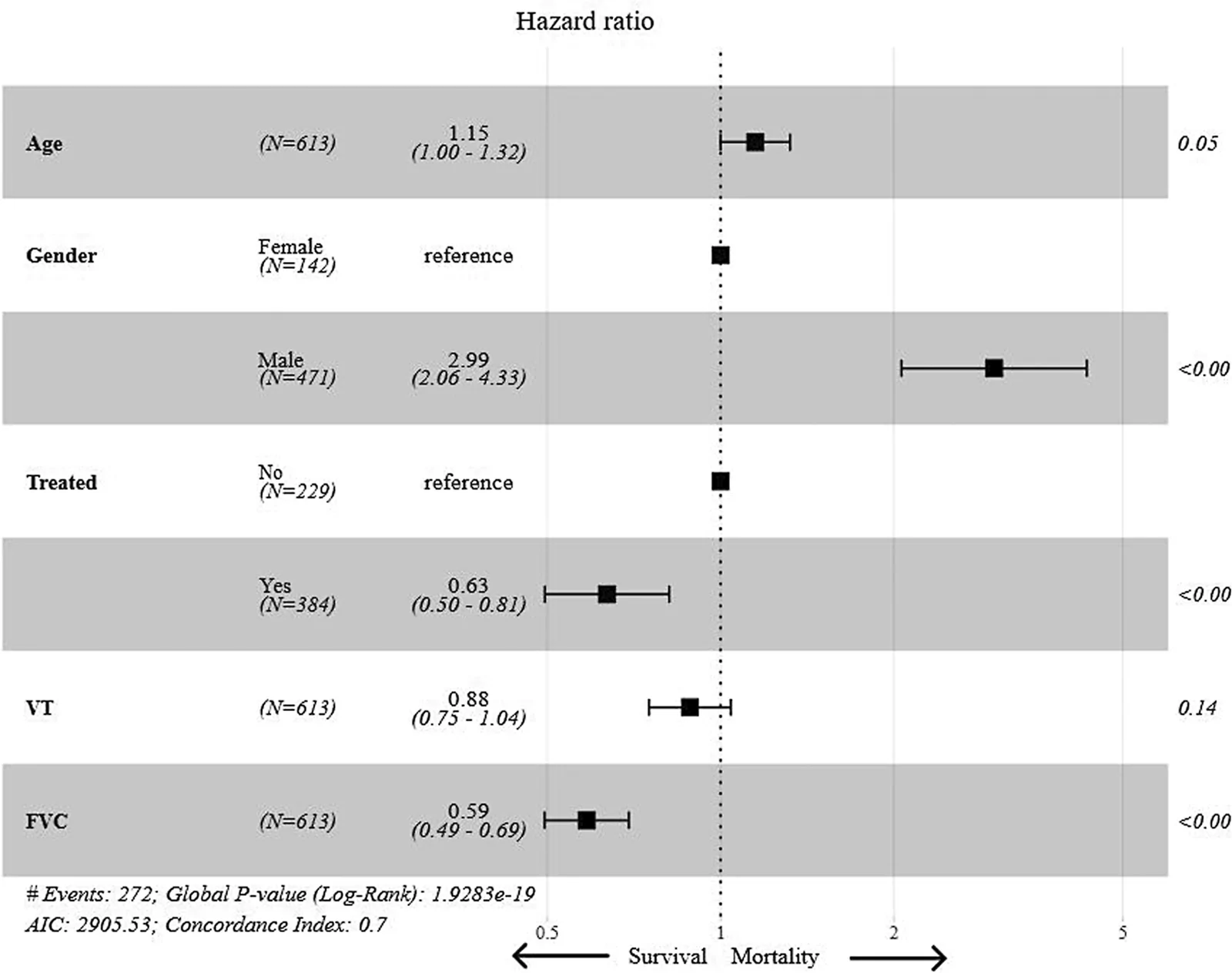
Multivariable Cox model: adjusted HRs for age, sex, antifibrotic treatment, tidal volume, forced vital capacity, and the VT × FVC interaction. Forest plot of multivariable Cox regression showing hazard ratios with 95% confidence intervals on a logarithmic scale for age, sex, VT, FVC, the VT × FVC interaction, and antifibrotic therapy in predicting mortality in IPF. The vertical reference line at HR = 1 indicates no effect. FVC, forced vital capacity; HR, hazard ratio; VT, tidal volume.

Figure 5 summarises the multivariable Cox proportional hazards model in a forest plot, displaying HRs with 95% confidence intervals on a logarithmic scale. In addition to age, sex, antifibrotic treatment, VT, and FVC at baseline, we also included the VT × FVC interaction in our model. VT and FVC were log-transformed and standardised due to their skewed distributions; the other covariates entered without transformation. Among the candidate model specifications, this model had the best Akaike information criterion.

**Fig. 6.**
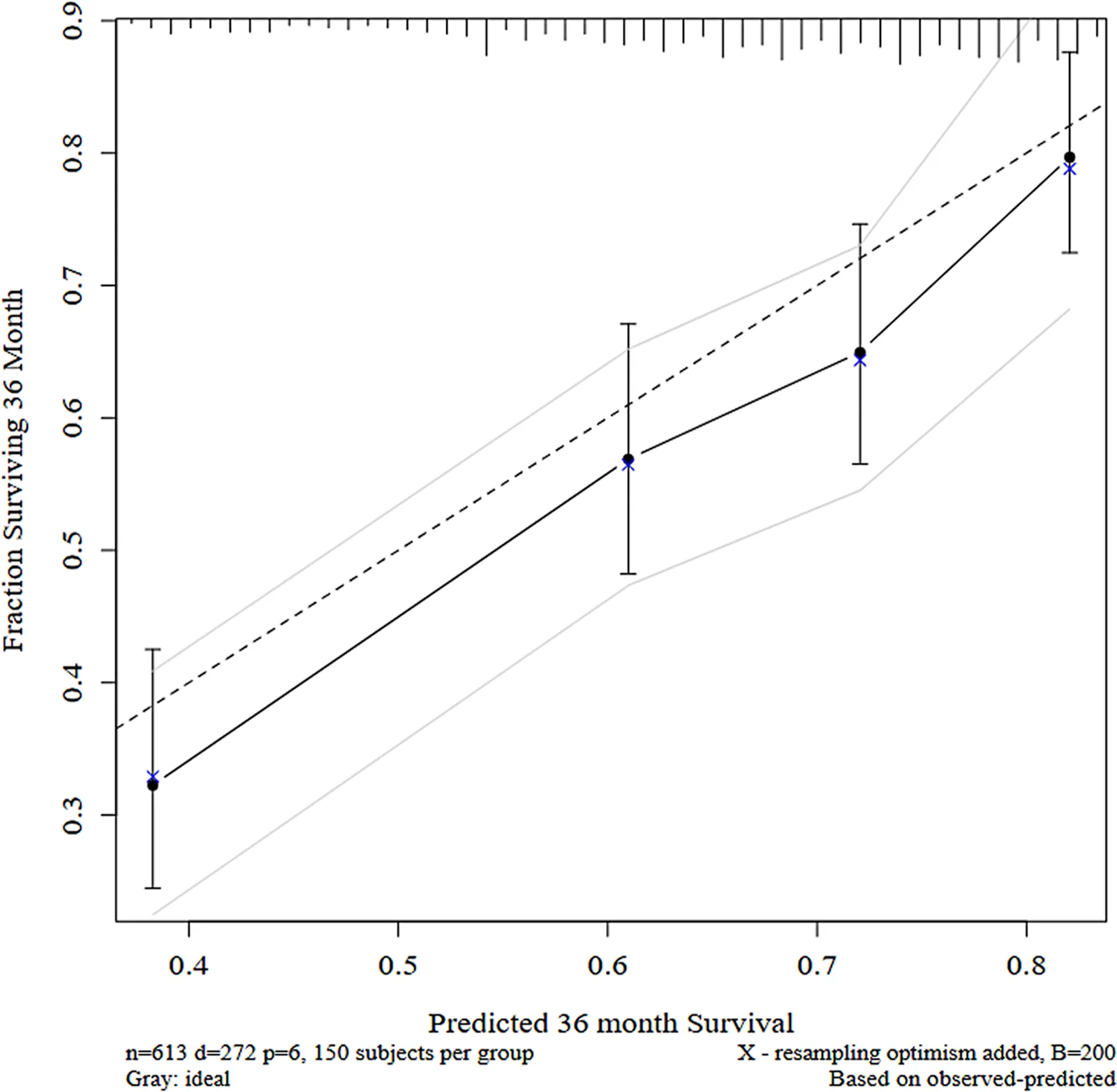
Calibration of the multivariable Cox model at 36 months. Calibration plot of the Cox regression model with age, sex, VT, FVC, the VT × FVC interaction, and antifibrotic therapy as covariates, evaluated at the 36-month time point. The diagonal reference line represents perfect calibration. FVC, forced vital capacity; VT, tidal volume.

Age showed a borderline association with mortality (HR: 1.15 per 10-year increment, 95% CI: 1.00–1.32; *p* = 0.05). Male sex was associated with the largest increase in mortality risk (HR: 2.99, 95% CI: 2.06–4.33; *p* < 0.001). FVC was strongly associated with outcome, with higher values conferring a substantial survival advantage (HR: 0.59, 95% CI: 0.49–0.69; *p* < 0.001), consistent with its central role as a marker of restrictive disease severity. VT showed a trend toward improved survival that did not reach statistical significance (HR: 0.88, 95% CI: 0.75–1.04; *p* = 0.14), and the VT × FVC interaction approached but did not reach significance (HR: 1.12, 95% CI: 0.98–1.30; *p* = 0.08). Antifibrotic therapy was associated with a statistically significant reduction in mortality risk (HR: 0.63, 95% CI: 0.50–0.81; *p* < 0.001). The calibration plot corresponding to this model is shown in Figure 6.

### Concordance Analysis

**Fig. 7.**
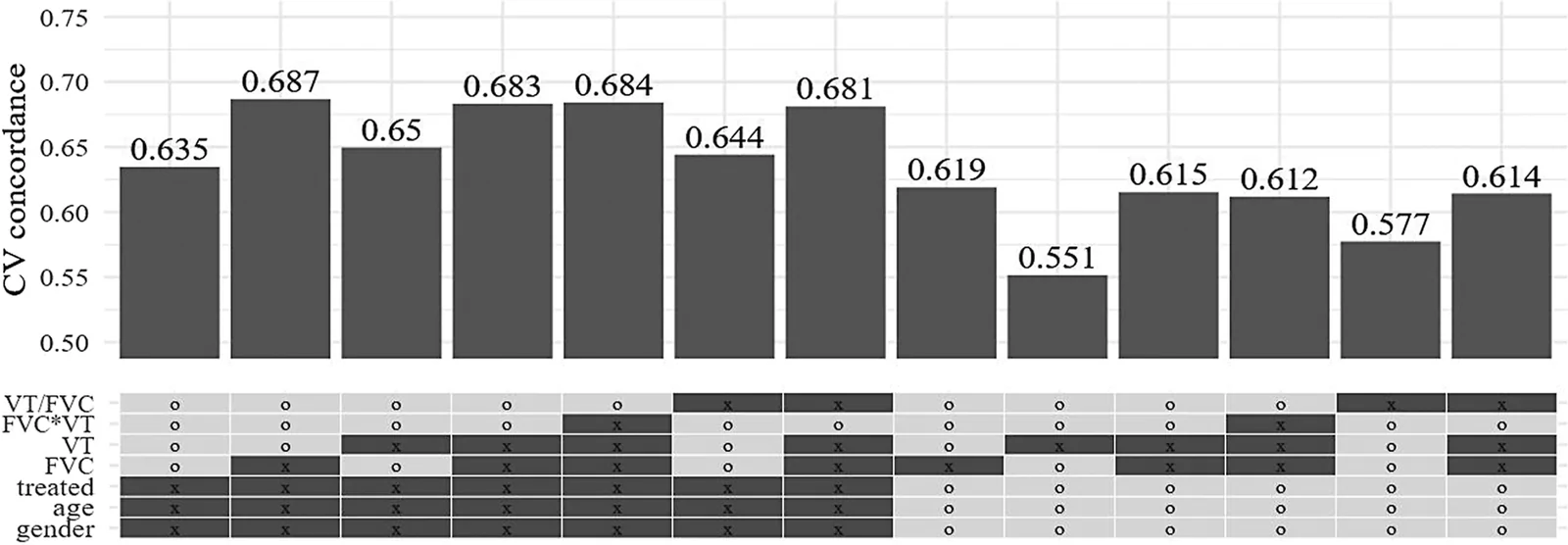
Impact of forced vital capacity and tidal volume on Cox model concordance for survival prediction. Cross-validated and apparent Harrell’s C-indices are shown for each pre-specified Cox model, demonstrating that adding FVC and/or VT to the base model improves predictive performance, with FVC contributing the most. FVC, forced vital capacity; VT, tidal volume.

Discriminative performance of the pre-specified Cox models is summarised in Figure 7. Apparent C-indices were 0.635 for the base model (age, sex, antifibrotic therapy), 0.650 with VT added, 0.687 with FVC added, 0.683 with both VT and FVC, and 0.684 with the addition of the VT × FVC interaction. Univariable C-indices were 0.551 for VT alone and 0.619 for FVC alone. Twenty-fold cross-validated C-indices were essentially unchanged (base: 0.634; base + FVC: 0.687; base + FVC + VT: 0.683; VT alone: 0.550), indicating limited optimism in the apparent estimates. Across all models, those including FVC consistently outperformed those without; the incremental gain from adding VT or the VT × FVC interaction on top of FVC was small (ΔC ≤0.005).

**Fig. 8.**
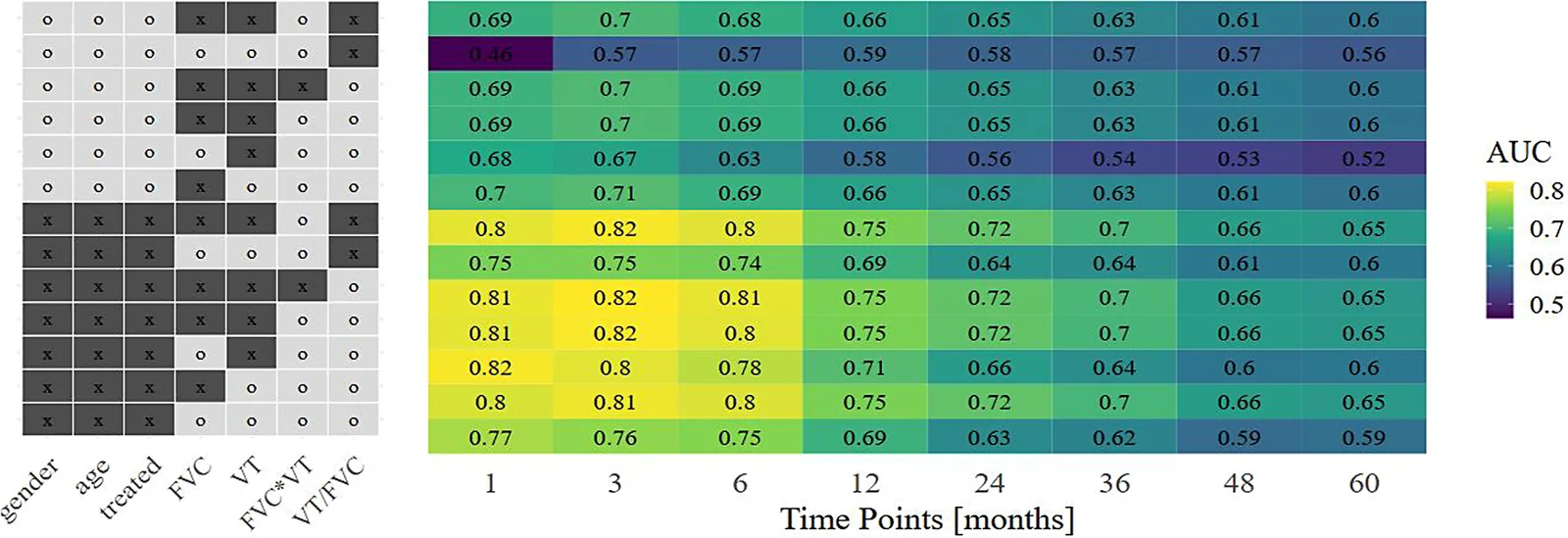
Time-dependent area under the receiver operating characteristic curve for survival prediction models including clinical variables, forced vital capacity, tidal volume, and their interaction. Time-dependent AUC values are shown for the evaluated Cox models across follow-up at 1, 3, 6, 12, 24, 36, 48, and 60 months. Each value represents the discriminative performance of one model at a specific time point. Higher values indicate greater ability to distinguish between patients who survived and those who did not at the same time point. AUC, area under the receiver operating characteristic curve; FVC, forced vital capacity; VT, tidal volume.

Time-dependent AUCs at 1, 3, 6, 12, 24, 36, 48, and 60 months are shown in Figure 8. The VT-only model achieved AUCs of approximately 0.55–0.56 between 12 and 36 months; the FVC-only model achieved 0.62 over the same interval; models combining VT and FVC reached 0.62–0.63; and the full model reached approximately 0.68–0.69 between 12 and 36 months. Discrimination at very early (1 month) and very late (60 month) time points was lower across all models, reflecting the small number of events and the small number of patients still at risk at the extremes.

## Discussion

The principal finding of this analysis is that VT in IPF behaves as a dynamic prognostic marker rather than a static one. Across the disease course, VT rises transiently during the phase of steepest FVC decline, sustains an elevated level while structural capacity is contracting most rapidly, and then returns slowly toward baseline as further compliance loss accumulates. The VT/FVC ratio contextualises this behaviour by relating operating volume to remaining structural capacity and differentiates survivors from non-survivors by indexing the mechanical burden imposed on resting ventilation. Although overall prognosis remains predominantly driven by FVC, VT and the VT × FVC interaction carry complementary information about lung volume utilisation that is technically straightforward to obtain in routine spirometry. Together these observations identify VT and VT/FVC as effort-independent physiological descriptors of restrictive remodelling that enrich the conventional FVC-based assessment of IPF by adding a resting ventilatory dimension to the standard spirometric one.

The substantive contribution of this work lies in the shape of the VT trajectory and its temporal coupling to FVC. In IPF, parenchymal stiffening progressively raises the elastic work of breathing and alters resting ventilatory strategy [16]. The observation that VT rises during the phase of steepest FVC decline, sustains an elevated level while structural capacity is contracting most rapidly, and then returns toward baseline as compliance loss accumulates fits a ventilatory adaptation hypothesis: increased VT may serve as a transient lower cost strategy for maintaining minute ventilation, before the rising elastic load makes high-VT breathing unsustainable and the breathing pattern shifts toward smaller, more frequent breaths. While rising central respiratory drive and the rapid shallow breathing pattern of advanced restrictive disease have been described previously, the intermediate VT-augmentation phase identified here and the longitudinal coupling between VT, FVC, and VT/FVC across the full disease course have not been characterised in IPF.

The trajectories partition the follow-up period into physiologically distinguishable segments. In the earliest months of progression, FVC begins to fall while VT rises modestly, suggesting that an immediate shift in resting breathing pattern is detectable alongside the first measurable signature of restrictive remodelling. As FVC enters its steepest decline, VT continues to rise to its peak, indicating that the ventilatory adjustment is concurrent with rather than subsequent to vital capacity loss. Later, as VT begins to fall, the VT/FVC ratio continues to climb but with a shallower slope because both numerator and denominator are now moving in the same direction. The asymmetry of the VT trajectory, a relatively rapid rise followed by a substantially longer decline, is itself informative: it suggests that the ventilatory adaptation establishes quickly once restrictive load begins and erodes slowly as that load increases further.

The VT/FVC ratio is the only one of the 3 markers that remains monotonic across the full follow-up, which is the strongest physiological argument for the ratio as a single integrative descriptor. It absorbs the non-monotonic behaviour of VT and the decelerating decline of FVC into a continuous, unidirectional signal of restrictive progression. FVC quantifies the loss of structural capacity, VT characterises the operational response of the respiratory system to that loss, and VT/FVC integrates both dimensions, indexing how close patients operate to their mechanical ceiling under resting conditions. Conceptually, the ratio therefore quantifies ventilatory reserve consumption during quiet breathing rather than under imposed stress, providing a continuous descriptor of restrictive burden that complements FVC rather than competing with it.

In multivariable analysis, FVC carried the dominant prognostic signal, while a single baseline measurement of VT did not reach independent significance once FVC was accounted for. This pattern is interpretable in the light of the trajectory: VT is a dynamic variable whose physiological meaning depends on where on the disease curve a given patient is measured, so a single time-point measurement captures only one phase of an evolving adaptation. The borderline VT × FVC interaction is consistent with this reading and suggests that VT and FVC carry a joint physiological signal that the baseline-only formulation cannot fully express. The implication is methodological as much as physiological: time-updated or landmark analyses of VT/FVC against outcome, capable of capturing the marker’s trajectory rather than only its baseline value, are the natural next step and may reveal prognostic content that the present baseline-only model cannot address. The discriminative ceiling observed across all FVC-containing models is consistent with the modest incremental contribution that any additional baseline spirometric marker can be expected to make to the prediction of transplant-free survival in IPF.

Tidal breathing metrics have predicted adverse respiratory outcomes in other contexts, including infant wheeze and cystic fibrosis, but have not previously been linked to long-term outcomes in fibrotic ILD [17]. Miki et al. [18] demonstrated that the inspired-expired oxygen concentration difference, a marker of ventilatory efficiency during peak exercise, was a stronger predictor of survival than peak V′O_2_, PaO_2_ slope, or even FVC. Vainshelboim et al. [6] demonstrated that abnormalities in ventilatory pattern, rather than spirometric impairment alone, carry independent prognostic information. Tidal volume reserve, V′E/V′CO_2_, and V′E/V′O_2_ all predicted mortality, indicating that patients who approach mechanical limits during exercise have significantly worse outcomes. Parallels can be drawn to prior work demonstrating the prognostic relevance of total lung capacity (TLC) in IPF. As shown by Kishaba et al. and Li et al. [19, 20] similar to VT/FVC, baseline reductions in TLC have been shown to predict early disease progression and poorer response to antifibrotic therapy, reflecting restrictive burden beyond forced expiratory manoeuvres. These findings support the concept that static volume measures capturing global lung restriction, such as TLC or the relationship between tidal operating volume and structural capacity, provide complementary insight into disease behaviour and progression that may not be fully captured by FVC alone. The present analysis extends these observations to resting tidal breathing, where ventilatory adaptation can be characterised without the need for cardiopulmonary exercise testing, broadening the population in whom such measurement is feasible.

The strengths of this study include its large multicentre cohort and the integration of cross-sectional, longitudinal, and survival analyses, enabling a robust assessment of resting breathing behaviour across disease severity and time. Limitations include variability in VT measurement related to breathing pattern and equipment, absence of respiratory rate and minute ventilation data, and the observational design, which precludes causal inference. The longitudinal trajectories were summarised by population-level smoothing of per-patient monthly averages and do not explicitly model informative dropout, so the late-follow-up flattening reflects the slow-progressing subset that remains in the cohort. The present analysis evaluated VT and VT/FVC at baseline and longitudinally but did not perform time-updated or landmark survival analyses; such analyses are the natural next step and would be expected to clarify the prognostic content of the VT/FVC trajectory beyond its baseline value. These limitations further emphasise that VT should be interpreted as a contextual rather than deterministic marker.

## Conclusions

In summary, VT in IPF behaves as a dynamic marker of ventilatory adaptation to progressive restrictive load, rising during the steepest phase of FVC decline and returning toward baseline as the elastic load accumulates. The VT/FVC ratio integrates this rising ventilatory drive with the falling vital capacity, evolves monotonically across the disease course, and stratifies survival on univariable analysis. Together, VT and VT/FVC provide an effort-independent physiological dimension that enriches the FVC-based assessment of IPF and offers a route to a more complete characterisation of disease progression. Integrated with imaging, exercise physiology, and digital respiratory biomarkers, VT-based measures may refine longitudinal monitoring of IPF without displacing FVC as the central prognostic measure.

## Acknowledgments

We gratefully thank the RARE-ILD consortium and the German Center for Lung Research, as well as all participating patients and physicians who contributed data to the registry and supported the continuation of this effort.

## Statement of Ethics

The eurIPFreg and eurILDreg study protocols were reviewed and approved by the Ethics Committees: Ethics Committee, Justus-Liebig University of Giessen, Germany – AZ 111/08; Ethics Committee, University General Hospital of Alexandroupolis, Greece – 38298/24-07-2014; Ethics Committee, Hospital Universitari de Bellvitge, Spain – 03/22; Ethics Committee, University of Catania, Italy – 123/2022; Ethics Committee, Medical University Vienna, 1045/2014; and Ethics Committee, Université Paris Cité, CPP SUD-EST IV – N° 2023-A01014-41. All research was performed in accordance with the Declaration of Helsinki. Patients were enrolled only after providing written informed consent. The eurIPFreg is registered at ClinicalTrials.gov (NCT02951416), eurILDreg – DRKS00028968.

## Conflict of Interest Statement

The other authors declare no conflicts of interest in regard to this study. M.M.M. declares receiving grants and fees for scientific advice, outside of this work, from Boehringer Ingelheim, Ferrer, Savara, and Insmed.

## Funding Sources

The European IPF Registry (eurIPFreg) was initially funded by the European Commission (FP7 European IPF Network) and has subsequently been supported through institutional funding, limited industry contributions (Roche and Boehringer Ingelheim), and the Lung Fibrosis Foundation Waldhof-Elgershausen, the German Lung Foundation, and the Robert Pfitzer Foundation. Its successor, eurILDreg, has received support from the RARE-ILD consortium through the European Joint Programme on Rare Diseases (EJP-RD). No specific funding was received for the present study. The funding sources supporting the registry infrastructure had no role in the study design, execution or analysis, or in the manuscript conception, planning, writing, or the decision to publish.

## Author Contributions

Conceptualisation: E.K., S.T., T.K.S., and A.G. Methodology: E.K., A.G., and S.T. Formal analysis: K.W. and T.K.S. Data curation: K.W., T.K.S., M.M.M., and B.C. Investigation: A.T., M.M.M., and C.V. Resources and project administration: S.T., A.G., B.C., and C.V. Visualisation: K.W. and M.M.M. Writing – original draft: E.K., S.T., P.M., A.G., and K.W. Writing – review and editing: M.M.M., B.C., and C.V. Supervision: A.G., B.C., and C.V. Validation: E.K., S.T., K.W., and T.K.S. All authors contributed to drafting or critically revising the manuscript, approved the final version for publication, and agreed to be accountable for all aspects of the work, ensuring that questions related to accuracy or integrity are appropriately investigated and resolved.

## Data Availability

Data supporting the findings of this study are available through the eurILDreg repository via the Portal für Medizinische Datenmodelle (MDM-Portal).
